# Seroepidemiology of Toxocara Infection in Patients With Vision Impairment and Blindness in Durango, Mexico

**DOI:** 10.14740/jocmr2032w

**Published:** 2014-12-29

**Authors:** Cosme Alvarado-Esquivel, Jesus Hernandez-Tinoco, Luis Francisco Sanchez-Anguiano

**Affiliations:** a Biomedical Research Laboratory, Faculty of Medicine and Nutrition, Juarez University of Durango State, Avenida Universidad S/N, 34000 Durango, Mexico; b Institute for Scientific Research “Dr. Roberto Rivera-Damm”, Juarez University of Durango State, Avenida Universidad S/N, 34000 Durango, Mexico

**Keywords:** Case-control study, Epidemiology, Vision impairment, Risk factors, Seroprevalence, Toxocariasis

## Abstract

**Background:**

*Toxocara* infection is a cause of vision impairment and blindness. We aimed to determine the seroprevalence and correlates of *Toxocara* infection in patients suffering from vision impairment and blindness in Durango City, Mexico.

**Methods:**

Through a cross-sectional seroprevalence study, 204 patients with vision impairment and 19 blind patients were studied for the presence of anti-*Toxocara* IgG antibodies in Durango City, Mexico. Seroprevalence association with socio-demographic, housing, clinical, and behavioral characteristics of participants was also investigated.

**Results:**

Five (2.5%) of the 204 patients with vision impairment, and none of the 19 patients with blindness were positive for anti-*Toxocara* IgG antibodies. In total, five of the 223 (2.2%) patients were positive for anti-*Toxocara* IgG antibodies. Seropositivity to *Toxocara* was not associated with age, sex, educational level, socio-economic status, presence of underlying diseases or behavioral characteristics of the patients. In contrast, multivariate analysis showed that *Toxocara* seropositivity was associated with living in a house with soil floor (odds ratio (OR) = 11.14; 95% confidence interval (CI): 1.57 - 78.74; P = 0.01).

**Conclusions:**

This is the first report of *Toxocara* exposure in patients with vision impairment and blindness in Mexico, and of an association of *Toxocara* exposure with living in a house with soil floors. Results suggest a low *Toxocara* exposure in these patients in Durango, Mexico. The risk factor associated with *Toxocara* exposure identified in this study warrants for further investigation.

## Introduction

*Toxocara* infection is an important cause of vision impairment and blindness [1] and has a worldwide distribution [2-4]. Toxocariasis is a neglected disease caused by several species of *Toxocara* including *canis*, *cati* and other species [5]. There are several routes of *Toxocara* infection including ingestion of parasite eggs from soil contaminated with excrement shed by dogs and cats infected with *Toxocara* [6, 7], and by ingestion of *Toxocara* larvae from undercooked giblets [8, 9]. After infection, *Toxocara* disseminates in the body by the bloodstream towards eyes, lungs, liver, muscles, and central nervous system [2, 10]. The clinical spectrum of *Toxocara* infection varies from asymptomatic to severe disease [2, 3, 11]. Toxocariasis can be limited to affection of eyes and optic nerves [2]; however, permanent blindness may occur [12, 13].

Very little is known about the epidemiology of *Toxocara* infection in patients suffering from eye diseases in Mexico in general and there is lack of knowledge in the northern Mexican state of Durango in particular. In a recent study in Mexico City, researchers found *Toxocara* seropositivity in eight of 21 patients with ankylosing spondylitis and acute non-granulomatous anterior uveitis [14]. We are not aware of any study aimed to determine the magnitude of *Toxocara* infection in patients suffering from vision impairment and blindness in Mexico. Therefore, we sought to determine the seroprevalence and correlates of *Toxocara* infection in patients with vision impairment and blindness in Durango City, Mexico.

## Materials and Methods

### Study population

Through a cross-sectional study using serum samples from a recent *Toxoplasma gondii* serosurvey [15], we determined the prevalence of anti-*Toxocara* IgG antibodies in patients suffering from vision impairment or blindness. Inclusion criteria for the patients were: 1) patients suffering from vision impairment or blindness attending public settings: the Mexican Social Security Institute (IMSS), Secretary of Health (SS), and State Government (SG); 2) aged 8 years and older; and 3) who accepted to participate in the study. Socio-economic status, sex, and educational level were not restrictive criteria for enrollment. In total, 223 patients were included in the study, 104 of them attended a public ophthalmology center (SG), 100 attended a public hospital for ophthalmology consultations (IMSS), and 19 attended a school for blind people (SG). Patients included 140 females and 83 males aged 8 - 96 years old (mean 50.49 ± 21.10 years).

### Socio-demographic, housing, clinical and behavioral characteristics of patients

The general characteristics of the patients were obtained with the aid of a standardized questionnaire. Items of socio-demographic data included age, sex, educational level, occupation and socio-economic status. The variable occupation was divided into two subgroups focused on the presence or absence of occupational exposure to soil (gardening or agriculture). We obtained the housing conditions of the patients by using the criteria of Bronfman et al [16]. For this purpose, we obtained information about the crowding at home (number of persons and rooms in the house), type of flooring of the house, availability of drinkable water, and form of elimination of excreta. Clinical data explored included the diagnosis of vision impairment, presence of underlying diseases, history of surgeries, blood transfusions or transplantations, presence of treatment, response to treatment, and presence of other impairments (in memory, reflexes or hearing). Behavioral data included contact with cats and dogs, foreign traveling, consumption of meat (pork, beef, goat, lamb, boar, chicken, turkey, pigeon, duck, rabbit, venison, squirrel, horse, opossum, or other), raw or undercooked meat and dried or processed meat (ham, sausages or chorizo), unwashed raw vegetables and fruits, drinking untreated water or unpasteurized milk, and frequency of eating away from home (in restaurants or fast food outlets).

### Laboratory tests

Sera from participants were kept frozen at -20 °C until analyzed. We determined the presence of anti-*Toxocara* IgG antibodies in the sera with a commercially available enzyme immunoassay “*Toxocara*” kit (Diagnostic Automation, Inc., Calabasas, CA, USA). The cut off for seropositivity was an absorbance reading equal to or greater than 0.3 optical density units. We included negative and positive controls in each assay and performed the tests following the manufacturer’s instructions.

### Statistical analysis

We used the software Epi Info version 7 and SPSS version 15.0 (SPSS Inc., Chicago, IL, USA) to analyze the data. For calculation of the sample size, we used a 95% confidence level, a reference seroprevalence of 13% [13] as the expected frequency of exposure in controls, and 5% of confidence limits. The result of the sample size calculation was 164 patients. The Pearson’s Chi-squared test and the two-tailed Fisher’s exact test (when values were small) were used to assess the association between *Toxocara* seropositivity and the socio-demographic, housing, clinical and behavioral characteristics of the patients. The association of *Toxocara* infection with the characteristics of the patients was further analyzed by multivariate analysis. As a strategy to include variables in the multivariate analysis, we selected variables with a P value < 0.10 obtained in the bivariate analysis. Odds ratios (ORs) and 95% confidence intervals (CIs) were calculated by means of the backward stepwise regression method. We used the Hosmer-Lemeshow goodness of fit test to assess the fitness of our regression model. Statistical significance was set at a P value less than 0.05.

### Ethical aspects

In this study, only archival serum samples and questionnaires from a previous survey [15] were used. The Institutional Ethical Committee of the Mexican Institute for Social Security in Durango City, Mexico approved this previous survey. In such survey, the purpose and procedures of the study were explained to all participants and written informed consent was obtained from all of them and from the next of kin of minor participants.

## Results

Five (2.5%) of the 204 patients with vision impairment and none of the 19 patients with blindness were positive for anti-*Toxocara* IgG antibodies. In total, five of the 223 (2.2%) patients with vision impairment or blindness were positive for anti-*Toxocara* IgG antibodies.

None of the socio-demographic characteristics of patients including age, sex, educational level, occupational contact with soil or socio-economic status showed P values < 0.10 by bivariate analysis (Table 1). With respect to the housing conditions of the patients, the variable “living in a house with soil floor” and “education of the head of the family” showed P values < 0.10. Other housing variables including crowding at home, availability of drinkable water, and form of elimination of excreta showed P values > 0.10. Of the clinical characteristics, seropositivity to *Toxocara* was not associated with the presence of underlying diseases, history of surgery, transplantation or blood transfusion, response to treatment, and presence of hearing, reflexes, or memory impairments.

**Table 1:** Socio-Demographic Characteristics of the Patients Studied

Characteristic	No. of patients studied	Prevalence of *Toxocara* infection		P value
		No.	%	
Age groups (years)				
≤ 30	25	1	4.0	0.3
31 - 50	69	0	0.0	
> 50	129	4	3.1	
Gender				
Male	83	3	3.6	0.36
Female	140	2	1.4	
Birth place				
Durango State	200	4	2.0	0.42
Other Mexican State or abroad	23	1	4.3	
Residence area				
Urban	158	3	1.9	0.53
Suburban	16	1	6.3	
Rural	48	1	2.1	
Educational level				
No education	18	0	0.0	0.58
1 - 12 years	184	5	2.7	
> 12 years	21	0	0.0	
Occupational exposure to soil				
Yes	35	2	5.7	0.25
No	181	2	1.1	
Socio-economic level				
Low	89	3	3.4	0.94
Medium	73	2	2.7	
High	2	0	0.0	

Of the behavioral characteristics of patients, the variables consumption of untreated water, and eating out of home showed P values < 0.10 (Table 2). Other behavioral characteristics of patients including contact with cats and dogs, foreign traveling, consumption of any type of meat, unwashed raw vegetables and fruits, and drinking unpasteurized milk showed P values > 0.10. Multivariate analysis of behavioral and housing characteristics with P values < 0.10 obtained by bivariate analysis showed that *Toxocara* seropositivity was associated only with the variable “living in a house with soil floor” (OR = 11.14; 95% CI: 1.57 - 78.74; P = 0.01). The result of the Hosmer-Lemeshow test (P = 0.99) indicated an acceptable fit of our regression model.

**Table 2:** Bivariate Analysis of Selected Behavioral and Housing Characteristics in Patients and Seroprevalence of *Toxocara* Infection

Characteristic	No. of patients tested*	Prevalence of *Toxocara* infection		P value
		No.	%	
Cats at home				
Yes	113	3	2.7	1.00
No	110	2	1.8	
Dogs at home				
Yes	92	2	2.2	1.00
No	131	3	2.3	
Frequency of meat consumption				
Never	2	0	0.0	0.14
1 or 2 times a week	126	5	4.0	
3 or more times a week	95	0	0.0	
Degree of meat cooking				
Undercooked	16	0	0.0	1.00
Well done	206	5	2.4	
Consumption of raw cow milk				
Yes	133	2	1.5	0.39
No	90	3	3.3	
Consumption of unwashed vegetables				
Yes	20	1	5.0	0.37
No	203	4	2.0	
Consumption of untreated water				
Yes	82	4	4.9	0.06
No	141	1	0.7	
Frequency of eating out of home				
Up to 10 times a year	120	5	4.2	0.06
> 10 times a year	103	0	0.0	
Availability of water				
In house	189	4	2.1	0.22
In land	9	1	11.1	
In street	5	0	0.0	
Sewage in house				
Yes	181	4	2.2	0.42
No	21	1	4.8	
Crowding at home				
No	112	3	2.7	0.28
Crowded	79	1	1.3	
Overcrowded	11	1	9.1	
Education of the head of family				
4 or more years	133	0	0.0	0.003
Up to 3 years	64	5	7.8	
Soil floor at home				
Yes	17	3	17.6	0.003
No	205	2	1.0	

*Patients with available data.

## Discussion

Very little is known about the seroepidemiology of *Toxocara* infection in Mexico. To the best of our knowledge, there is not any study about the seroepidemiology of *Toxocara* infection in patients suffering from vision impairment or blindness in Mexico. The present serosurvey was performed to investigate the seroprevalence and correlates of *Toxocara* exposure in these patients in the northern Mexican city of Durango. Results of the present study suggest a low (2.2%) seroprevalence of *Toxocara* exposure in patients with blindness or vision impairment. The seroprevalence found in patients with vision impairment or blindness is lower than those (4.7-26.2%) reported in other population groups in the region. A 26.2% seroprevalence of *Toxocara* exposure was found in the ethnic group of Tepehuanos in rural Durango, Mexico [17]. In a study of waste pickers in Durango City, a seroprevalence of 13% was found [18]. In addition, patients in a psychiatric hospital in the same city had a 4.7% seroprevalence of *Toxocara* infection [19]. However, comparison of these seroprevalences should be interpreted with care because differences in ages in populations among the studies exist. The mean age in patients with vision impairment or blindness was 50.49 years old, while mean age in Tepehuanos was 32.46 years old, in waste pickers was 36.0 years old, and in psychiatric patients was 43.57 years old. Furthermore, differences in sanitation and hygiene practices might explain the differences in *Toxocara* seroprevalences among the population groups. It is likely that most patients with vision impairment or blindness had had better sanitation and hygiene practices than waste pickers, rural Tepehuanos and psychiatric patients.

In the present study, none of the socio-demographic characteristics of the patients including age, sex, educational level, or socio-economic status was associated with *Toxocara* exposure. Our results are consistent with those reported in a recent study where researchers found no association of the presence of anti-*Toxocara* antibodies with age, gender, and socio-economic status in healthy adults in southeast Brazil [20]. Contact with cats and dogs might represent a risk for *Toxocara* infection; however, we did not find an association of infection and contact with cats or dogs. This result also agrees with the lack of association between these variables in the study of healthy adults in Brazil [20]. An important risk factor for *Toxocara* infection is contact with soil [6, 7, 20]. In our studied population, patients with occupational exposure to soil had a higher seroprevalence of *Toxocara* infection than those without occupational exposure to soil. However, the difference in seroprevalence among these groups was not statistically significant. It is likely that the low number of *Toxocara*-seropositive patients found did not allow obtaining a statistically significant difference in the seroprevalences among the groups. Interestingly, multivariate analysis showed an association of *Toxocara* exposure with living in a house with soil floors. To the best of our knowledge, this is first report of this association. Soil floors at home that are contaminated with excrement of *Toxocara*-infected cats or dogs may contribute to transmit *Toxocara* infection to humans. The fact that *Toxocara* infection was associated with soil floors at home but not with occupational exposure to soil suggests that patients had more risk to acquire *Toxocara* infection at home than at work. Further studies should consider searching *Toxocara* in soil floors at homes. In addition, people living in houses with soil floors and having cats or dogs should consider taking preventive measures against *Toxocara* infection, for instance, deworming of their pets. However, soil flooring at home is a reflection of poverty and people living in this disadvantaged condition cannot afford to deworm their pets. Therefore, governmental actions to aid people living in poverty for changing soil floors with concrete floors at homes and deworming of their pets are highly needed.

### Conclusions

This is the first report of *Toxocara* exposure in patients with vision impairment and blindness in Mexico, and of an association of *Toxocara* exposure with living in a house with soil floors. Results suggest a low *Toxocara* exposure in these patients in Durango, Mexico. The risk factor associated with *Toxocara* exposure identified in this study deserves further investigation.
